# Role of Synovial Exosomes in Osteoclast Differentiation in Inflammatory Arthritis

**DOI:** 10.3390/cells10010120

**Published:** 2021-01-10

**Authors:** Ji Eun Song, Ji Soo Kim, Ji Hye Shin, Ki Won Moon, Jin Kyun Park, Kyong Soo Park, Eun Young Lee

**Affiliations:** 1 Department of Molecular Medicine and Biopharmaceutical Sciences, Graduate School of Convergence Science and Technology, Seoul National University, Seoul 08826, Korea; clara_1202@naver.com (J.E.S.); kspark@snu.ac.kr (K.S.P.); 2 Department of Family Medicine, Seoul National University Hospital, Seoul 03080, Korea; clarekim89@gmail.com; 3 Division of Rheumatology, Department of Internal Medicine, Seoul National University College of Medicine, Seoul 03082, Korea; kidjihye@gmail.com (J.H.S.); jinkyunpark@gmail.com (J.K.P.); 4 Division of Rheumatology, Department of Internal Medicine, Kangwon National University Hospital, Chuncheon 24289, Korea; ismoon1975@naver.com; 5 Division of Endocrinology, Department of Internal Medicine, Seoul National University College of Medicine, Seoul 03082, Korea

**Keywords:** synovial exosomes, osteoclastogenesis, inflammatory arthritis

## Abstract

This study aimed to investigate the characteristics of exosomes isolated from synovial fluid and their role in osteoclast differentiation in different types of inflammatory arthritis. Exosomes isolated from synovial fluid of rheumatoid arthritis (RA), ankylosing spondylitis (AS), gout, and osteoarthritis (OA) patients were co-incubated with CD14+ mononuclear cells from healthy donors without macrophage colony-stimulating factor (M-CSF) and receptor activator of nuclear factor kappa-B ligand (RANKL). Osteoclast differentiation was evaluated via tartrate-resistant acid phosphatase (TRAP) staining and activity and F-actin ring formation. RANKL expression on synovial exosomes was assessed using flow cytometry and an enzyme-linked immunosorbent assay (ELISA). Synovial exosomes were the lowest in OA patients; these induced osteoclastogenesis in the absence of M-CSF and RANKL. Osteoclastogenesis was significantly higher with more exosomes in RA (*p* = 0.030) than in OA patients, but not in AS or gout patients. On treating macrophages with a specified number of synovial exosomes from RA/AS patients, exosomes induced greater osteoclastogenesis in RA than in AS patients. Synovial exosomal RANKL levels were significantly higher in RA (*p* = 0.035) than in AS patients. Synovial exosome numbers vary with the type of inflammatory arthritis. Synovial exosomes from RA patients may bear the disease-specific “synovial signature of osteoclastogenesis.”

## 1. Introduction

Inflammatory arthritis (IA) involves the immune system and is characterized by joint damage and synovial inflammation; types of inflammatory arthritis include rheumatoid arthritis (RA), ankylosing spondylitis (AS), psoriatic arthritis, gout, and systemic lupus erythematosus (SLE) [1]. Synovial fluid from IA patients contains numerous immune cells, such as macrophages, B lymphocytes, T lymphocytes, and neutrophils, which produce numerous pro-inflammatory cytokines and proteolytic enzymes with roles in immune responses and bone destruction [2,3]. Furthermore, IA is characterized by different bone remodeling patterns [4,5,6]. In RA and gout, joint damage is characterized by extensive bone destruction resulting from osteoclast differentiation [7]; however, in AS, bone remodeling predominantly results from consecutive osteogenesis. 

Osteoclasts are bone-resorbing multinucleated cells that differentiate from the monocyte/macrophage cell lineage. Macrophage-colony stimulating factor (M-CSF) and receptor activator of nuclear factor NF-κB (RANKL) are essential for osteoclast differentiation [8]. Furthermore, inflammatory cytokines, such as TNF-α, IL-1 [9,10], IL-32 [11], and IL-33 [12], can stimulate osteoclastogenesis via a RANKL-independent pathway. Osteoclasts express tartrate-resistant acid phosphatase (TRAP), dendritic cell-specific transmembrane protein (DC-STAMP), cathepsin K (CTSK), and β3-intergrin for the cellular fusion of osteoclast precursors and their differentiation into mature osteoclasts [13].

Exosomes are endosome-derived membrane vesicles (40–200 nm) released by most cell types and mediate intercellular communication [14]. Exosomes transfer cellular components, such as proteins, microRNAs, mRNAs, and lipids, to recipient cells and contribute to intracellular communication after internalization of recipient cells and are present in various biological fluids, including blood, urine, amniotic fluid, saliva, malignant ascites, and synovial fluid [15,16]. Leukocyte-derived microparticles from RA patients induce proteolytic enzymes, such as matrix metalloproteinase 1 (MMP1), MMP3, MMP9, and MMP13, and pro-inflammatory cytokines, such as IL-6, IL-8, MCP-1, and MCP-2, via stimulation of synovial fibroblasts [17]. Furthermore, high levels of the long non-coding RNA HOTAIR (HOX transcript antisense intergenic RNA) have been reported in serum-derived exosomes of RA patients. Exosomes containing HOTAIR induce the migration of activated macrophages [18]. Interestingly, CD3+ and CD8+ T cell-derived microvesicles levels are increased in the plasma of RA patients compared to those in osteoarthritis (OA) patients, and RANKL-positive microvesicles are present in the biological fluids of RA patients [19].

Plasma-derived exosomes from multiple myeloma patients have recently been reported to induce osteoclast differentiation in murine RAW264.7 cells and human primary pre-osteoclasts [20]. Moreover, non-small cell lung cancer (NSCLC)-derived exosomes can potentially induce osteoclastogenesis through activation of the epidermal growth factor receptor (EGFR) pathway [21].

In addition, synovial fluid of IA patients, including RA and pyrophosphate arthropathy, contain high levels of TNF-α and are characterized by enhanced RANKL-induced osteoclastogenesis and resorption [22]. Inflammatory cytokines, such as IL-1β, TNF-α, and IL-8, are upregulated in the synovial fluid in IA patients [23,24], and these inflammatory cytokines, including TNF-α [25,26], IL-1 [27,28], and IL-6 [29], stimulate osteoclast differentiation. However, the molecular mechanisms underlying different patterns of bone remodeling in various types of IA are unclear. Therefore, we sought to characterize exosomes from the synovial fluid of various active IAs and determine whether synovial fluid-derived exosomes are involved in synovial fluid-mediated bone destruction. 

## 2. Materials and Methods

### 2.1. Preparation of Human Synovial Fluid

Synovial fluid was aspirated from knee joints of patients with RA (*n* = 20), AS (*n* = 7), gout (*n* = 8), and OA (*n* = 10) in accordance with the American College of Rheumatology criteria [30]. Samples were obtained from outpatients of Seoul National University Hospital and Kangwon National University Hospital after obtaining written informed consent in accordance with the tenets of the Declaration of Helsinki. The study was approved by the institutional review board of the Seoul National University Hospital [IRB-No 1603-146-751]. For further analysis, synovial fluid samples were centrifuged at 3000 rpm for 10 min to eliminate cells and frozen at −20 °C until use. The synovial fluid samples were treated with 2 μg/mL hyaluronidase (Sigma-Aldrich, St. Louis, MO, USA) for 1 h at 25 °C and centrifuged at 10,000× *g* for 10 min to eliminate debris, followed by exosome isolation. 

### 2.2. Exosome Characterization

#### 2.2.1. Exosome Isolation

Exosomes were isolated from the synovial fluid using the ExoQuickTM exosome precipitation solution (System Biosciences, Mountain View, CA, USA) in accordance with the manufacturer’s instructions. Hyaluronidase-treated synovial fluid samples were centrifuged at 10,000× *g* for 15 min at 4 °C to eliminate debris. Thereafter, 250 μL of synovial fluid was mixed with 63 μL of ExoQuick solution and incubated at 4 °C overnight. The ExoQuick-synovial fluid mixture was centrifuged at 1500 × for 30 min at 4 °C to obtain exosome pellets, which were resuspended in 1:10 of synovial fluid volume, using distilled water or PBS.

#### 2.2.2. Transmission Electron Microscopy (TEM) 

Ten microliters of each exosomal suspension was placed on a 200 mesh formvar-coated copper grid and negatively stained with 10 μL of 2% phosphotungstic acid (PTA). Images were analyzed using a JEM 1010 (JEOL, Tokyo, Japan) transmission electron microscope operated at 80 kV.

#### 2.2.3. Size Distribution

The size distribution of exosomes was determined using a Nanosight LM10 (Malvern Instruments, Malvern, UK). Exosome pellets were resuspended in 1 mL of distilled water and analyzed using the Nanoparticle Tracking Analysis 3.1 (NTA) software. 

#### 2.2.4. Exosome Quantification 

To determine the number of synovial exosomes, acetylcholinesterase (AChE) activity and CD81 expression were evaluated using the EXOCET exosome quantitation assay (System Biosciences) and CD81 ExoELISA (System Biosciences) in accordance with the manufacturer’s instructions. Since AChE is enriched within exosomes [31] and CD81 is an exosomal surface marker [32], their activity and expression levels indirectly determined the number of exosomes. 

#### 2.2.5. Exosome Labeling and Uptake

Synovial exosomes were labeled with carboxyfluorescein succinimidyl diacetate ester (CFSE) using Exo-Green (System Biosciences) in accordance with the manufacturer’s instructions. Labeled exosomes were incubated with macrophages for 3 h at 37 °C. Cells were fixed with 3.7% formaldehyde, and nuclei were stained with 2 μg/mL Hoechst 33,258 (Sigma–Aldrich, St. Louis, MO, USA) for 5 min at 25 °C. Finally, cellular uptake of CFSE-labeled exosomes was analyzed using a Leica TCS SP8 microscope (Leica, Wetzlar, Germany).

#### 2.2.6. RANKL-ELISA

RANKL levels in synovial exosomes were determined using a RANKL ELISA Kit (Cusabio, Hubei, China). Exosome pellets isolated from 400 μL of synovial fluid obtained from RA (*n* = 7) and AS (*n* = 6) patients were resuspended in 320 μL of lysis buffer, followed by sonication using a bioruptor (Cosmo bio, Tokyo, Japan). For further lysis, samples were incubated for 20 min at 37 °C and centrifuged at 13,000 rpm for 10 min to eliminate debris. RANKL levels in lysed exosomes were determined in accordance with the manufacturer’s instructions. 

#### 2.2.7. Flow Cytometry

RANKL expression on the isolated synovial exosomes from RA and AS patients was assessed via flow cytometry using ExoFlow (System Biosciences) in accordance with the manufacturer’s instructions. The magnetic streptavidin Exo-Flow beads were coated with biotinylated anti-CD9 (clone SN4 C3-3A2; eBioscience, San Diego, CA, USA) and RANKL (R&D systems, Minneapolis, MN, USA) capture antibodies. Synovial exosomes isolated from 500 μL of synovial fluid were incubated with CD9 or RANKL-conjugated beads overnight at 4 °C. Captured exosomes were stained with Exo-FITC exosome stain and analyzed using a FACSCalibur flow cytometer and Cellquest software (BD Biosciences, San Jose, CA, USA).

### 2.3. Exosome and Osteoclastogenesis

#### 2.3.1. In Vitro Exosome Functional Assays

Human blood samples were obtained from healthy volunteers at Seoul National University Hospital. Peripheral blood mononuclear cells (PBMCs) were isolated using Ficoll-PaqueTM PLUS (GE Healthcare Life Sciences, Chicago, IL, USA). Isolated PBMCs were magnetically labeled with CD14 MicroBeads (Miltenyi Biotec, Bergisch Gladbach, Germany), followed by positive selection. CD14+ monocytes were differentiated into macrophages via treatment with 20 ng/mL M-CSF (Miltenyi Biotec, Germany) for 4–6 days. Thereafter, macrophages were incubated with 10% synovial exosomes in alpha minimum essential medium (α-MEM; Gibco, Waltham, NY, USA) supplemented with 10% FBS and 1% penicillin/streptomycin for an additional 9–10 days. Synovial exosomes were isolated from the same volume of synovial fluid. To generate multinucleated osteoclasts as a positive control, cells were treated with 20 ng/mL M-CSF and 40 ng/mL RANKL (Miltenyi Biotec), while medium alone was used as a negative control. Half of the culture medium was replenished every alternate day. 

#### 2.3.2. Cell Proliferation Assay

To assess cell proliferation, a cell counting kit-8 (CCK-8) (Dojindo, Kumamoto, Japan) was added to synovial exosome-treated macrophages. After 2 h of incubation, optical density was measured spectrophotometrically at 450 nm.

#### 2.3.3. TRAP Staining and Activity

Osteoclastogenesis was evaluated through Tartrate-resistant acid phosphatase (TRAP) staining. TRAP expression, a marker for osteoclastogenesis [33], was detected using an Acid phosphatase kit (Sigma–Aldrich) in accordance with the manufacturer’s instructions. The number of TRAP-positive multinucleated cells containing more than three nuclei (cells/cm^2^) was determined through light microscopy. TRAP activity was measured using a TRACP & ALP Assay Kit (Takara, Shiga, Japan) in accordance with the manufacturer’s instructions. The absorbance of TRAP activity was analyzed at 405 nm and expressed as a fold change in the medium alone group.

#### 2.3.4. Evaluation of Actin Ring Formation

Cells were fixed with 3.7% formaldehyde in PBS for 20 min at 25 °C and then permeabilized with 0.1% Triton X-100 in PBS for 10 min at 25 °C. Cells were stained with 50 μg/mL phalloidin-FITC (Sigma–Aldrich) for 40 min at 25 °C, and nuclei were stained with 2 μg/mL Hoechst 33,258 (Sigma–Aldrich) for 5 min at 25 °C. F-actin rings were observed using a Leica DM5500B microscope. 

### 2.4. Statistical Analysis

Statistical analysis was performed using Mann–Whitney U tests, using SPSS Statistics software V. 22.0 (IBM, Armonk, NY, USA). Data are presented as the mean ± standard error of the mean (SEM). *p*-values less than 0.05 were considered statistically significant.

## 3. Results

### 3.1. Characterization of Synovial Exosomes in Inflammatory Arthritis

**Figure 1:**
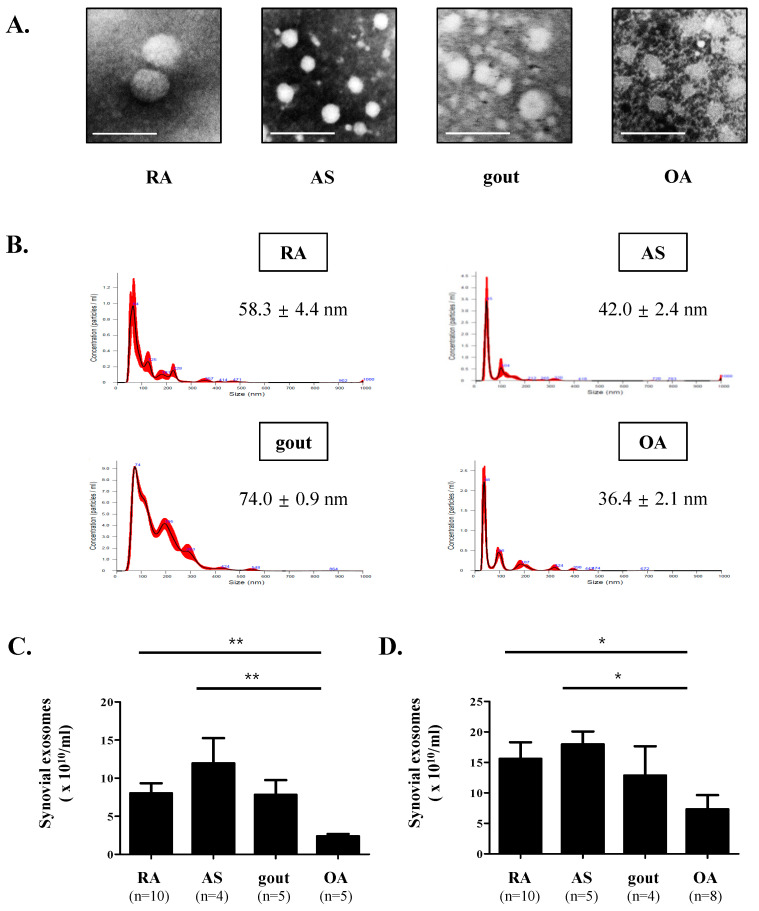
Characterization of synovial exosomes. (**A**) The shape and size of synovial exosomes purified from rheumatoid arthritis (RA), ankylosing spondylitis (AS), gout, and osteoarthritis (OA) patients were observed using a transmission electron microscope (TEM). The size of synovial exosomes ranged in diameter from 20 to 200 nm. Scale bar = 200 nm. (**B**) The size distribution of synovial exosomes was measured using a Nanosight LM 10 and analyzed with enzyme-linked immunosorbent assay (NTA) 3.1 software. Red error bars indicate +/−1 standard error of the mean, and the modal size of synovial exosomes are shown for each preparation. (**C**,**D**) The number of synovial exosomes was assessed by acetyl-CoA acetylcholinesterase (AChE) activity and CD81-ELISA. Data are presented as the mean ± SEM. * *p* < 0.05 vs. OA, ** *p* < 0.01 vs. OA.

The isolated exosomes were imaged using transmission electron microscopy (Figure 1A); they were sized between 20 and 200 nm, which is the expected size of exosomes [14]. 

We identified the modal size and number of synovial exosomes to be 58.3 ± 4.4 nm and 4.52 × 10^11^ ± 4.19 × 10^10^ particles/mL in RA patients; 42.0 ± 2.4 nm and 6.82 × 10^11^ ± 8.56 × 10^10^ particles/mL, AS; 74.0 ± 0.9 nm and 8.96 × 10^11^ ± 2.51 × 10^10^ particles/mL, gout; 36.4 ± 2.1 nm and 1.24 × 10^11^ ± 3.96 × 10^9^ particles/mL, OA, respectively (Figure 1B). Isolated synovial exosomes were nano-sized vesicles per the enzyme-linked immunosorbent assay (NTA) software, similar to TEM results.

As exosomes are highly enriched with AChE [31] and CD81 [32], their activity and expression levels indirectly reflect the number of synovial exosomes. An EXOCET assay revealed that the number of synovial exosomes was significantly higher in RA patients (8.033 ± 1.301 × 10^10^ particles/mL, *p* = 0.003) and in AS patients (11.95 ± 3.315 × 10^10^ particles/mL, *p* = 0.032) than in OA patients (2.396 ± 0.2835 × 10^10^ particles/mL) (Figure 1C). The number of synovial exosomes was slightly, but not significantly, higher in gout patients (7.806 ± 1.936 × 10^10^ particles/mL, *p* = 0.095) than in OA patients. 

Similarly, CD81 levels were significantly higher in RA patients (15.59 ± 2.722 × 10^10^ particles/mL, *p* = 0.043) and in AS patients (17.96 ± 2.107 × 10^10^ particles/mL, *p* = 0.030) than in OA patients (7.304 ± 2.324 × 10^10^ particles/mL) (Figure 1D). CD81 levels were slightly, but not significantly, higher in gout patients (12.86 ± 4.788 × 10^10^ particles/mL, *p* > 0.05) than in OA patients (7.304 ± 2.324 × 10^10^ particles/mL). These results indicate that synovial exosomes are significantly higher in IA including RA and AS than in non-IA including OA. 

We investigated whether synovial exosomes isolated from IA patients might induce osteoclastogenesis. To determine whether synovial exosomes are internalized by human macrophages (potential recipient cells), synovial exosomes were labeled with CFSE. Labeled exosomes co-incubated with macrophages localized in the perinuclear region after 3 h of incubation (Figure S1, Supplementary Materials). 

### 3.2. The Role of Exosomes on Osteoclastogenesis

**Figure 2:**
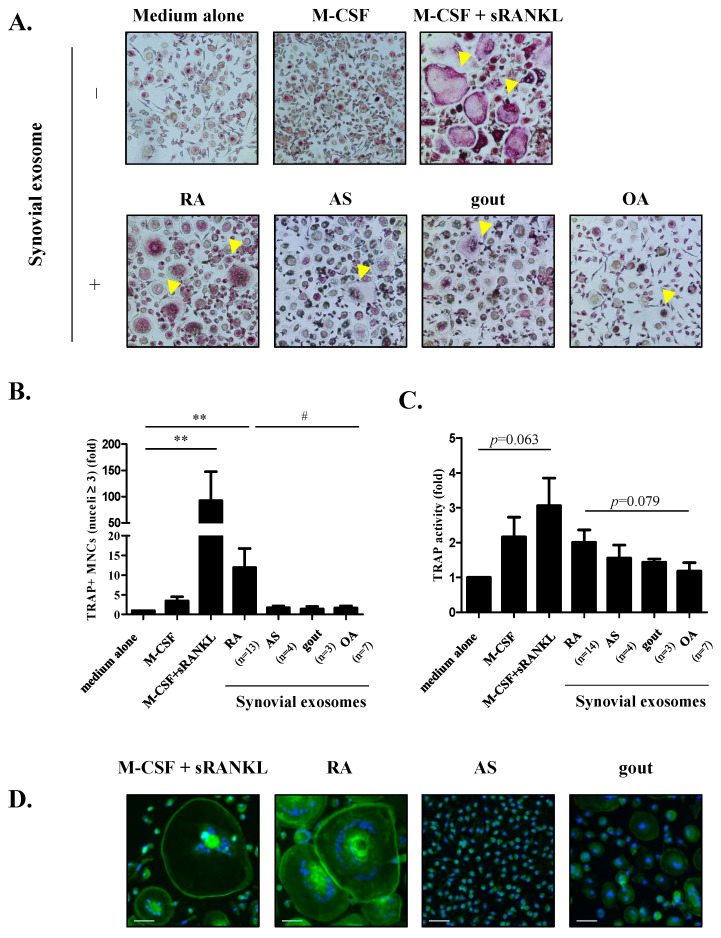
Treatment of synovial exosomes with human macrophages and the effect of synovial exosomes on human osteoclastogenesis. (**A**) Human macrophages differentiated from CD14+ monocytes were treated with 20 ng/mL of macrophage colony-stimulating factor (M-CSF) + 40 ng/mL of receptor activator of nuclear factor kappa-B ligand (RANKL) (positive control) or with 10% synovial exosomes, respectively. Exosomes were isolated from the same volume of synovial fluid with rheumatoid arthritis (RA), ankylosing spondylitis (AS), gout, and osteoarthritis (OA) patients. After 9–10 days, cells were stained for tartrate-resistant acid phosphatase (TRAP) expression, and TRAP-positive multinucleated cells (MNCs) were imaged using light microscopy. The yellow arrow indicates TRAP-positive MNCs. Magnification: ×100. (**B**) The number of TRAP-positive multinucleated (more than three) osteoclasts were counted (cells/cm^2^). Data are presented as a fold change in the osteoclast number compared to medium alone (negative control) and mean ± SEM. ** *p* < 0.01 vs. medium alone, # *p* < 0.05 vs. OA. (**C**) TRAP activity was measured at 405 nm and expressed as fold change in the medium alone. Data are presented as the mean ± SEM. (**D**) Actin rings were stained with FTIC-phalloidin, and nuclei were stained with Hoechst 33258. The white arrow indicates the F-actin ring. Scale bar = 50 μm.

To investigate whether synovial exosomes internalized into macrophages regulate osteoclast differentiation, TRAP staining was performed, and TRAP activity was analyzed. Human macrophages treated with synovial exosomes of RA, AS, gout, and OA patients differentiated into TRAP-positive multinucleated cells in the absence of M-CSF and RANKL (Figure 2A). Among them, exosomes isolated from RA patients had higher osteoclastogenic potential than AS, gout, and OA patients. Osteoclastogenesis was significantly increased in the presence of exosomes of RA patients (11.90 ± 4.873 folds, *p* = 0.030) than in those of OA patients (1.647 ± 0.5231 folds), but not in those of AS patients (1.703 ± 0.4481 folds) or gout patients (1.373 ± 0.7031 folds) (Figure 2B). Thereafter, we investigated the effect of synovial exosomes on TRAP activity. Macrophages treated with synovial exosomes of RA patients (2.011 ± 0.3548 folds, *p* = 0.079) displayed relatively higher TRAP activity than those of OA patients (1.183 ± 0.2440 folds), but less prominent in AS patients (1.559 ± 0.3734 folds) or gout patients (1.435 ± 0.09832 folds) (Figure 2C). In summary, these data indicate that synovial exosomes potentially induce osteoclast differentiation in RA.

Osteoclasts form an actin ring, known as the sealing zone, to compactly adhere with the bone surface. These structural changes allow for effective bone matrix resorption by osteoclasts [34]. Hence, we assessed osteoclastogenesis with F-actin rings to analyze resorptive activity in mature osteoclasts. Macrophages treated with synovial exosomes of RA patients differentiated into osteoclast-like cells with F-actin rings, similar to the positive control (Figure 2D); however, F-actin rings were rarely observed in those of AS and gout patients. These data suggest that synovial exosomes can induce osteoclastogenesis accompanied by bone matrix resorption through actin cytoskeletal rearrangement in RA.

To determine whether the effect of synovial exosomes on human osteoclastogenesis was related to cell proliferation, we assessed the proliferation of exosome-treated macrophages. Synovial exosomes induced cell proliferation; however, there were no significant differences among patients with RA, AS, gout, and OA (Figure S2, Supplementary Materials). The cell proliferation assay revealed no association between cell proliferation and osteoclast differentiation.

We sought to determine whether the differences in exosome quality affect osteoclast differentiation by normalizing the number of treated exosomes owing to differences in exosome number and quality depending on the disease. Hence, macrophages were treated with a specified number of synovial exosomes (7.59 × 10^9^ particles/mL) from RA and AS patients, similar in the number of synovial exosomes. Despite treatment with the same number of exosomes, exosomes of RA patients tended to induce slightly, but not significantly, greater osteoclastogenesis (89.87 ± 28.35 folds) than those of AS patients (47.00 ± 41.50 folds) (Figure S3, Supplementary Materials). Together, our results suggest that differences in exosome quality may affect osteoclastogenesis.

**Figure 3:**
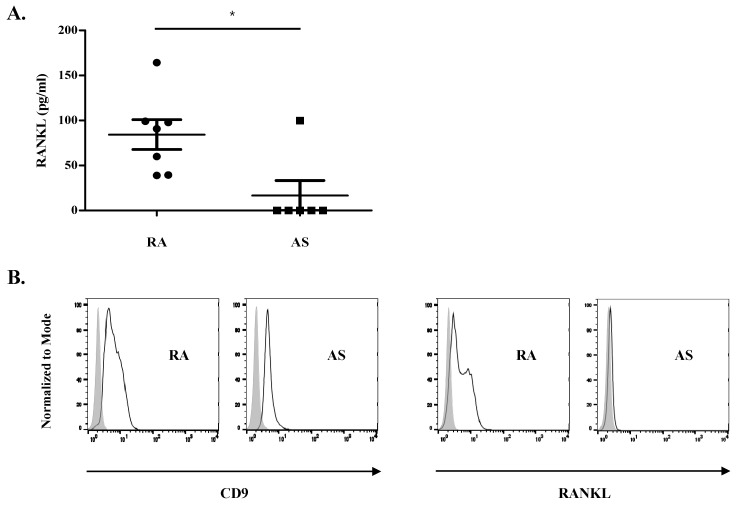
RANKL expression on synovial exosomes. (**A**) Exosomes from rheumatoid arthritis (RA) and ankylosing spondylitis (AS) patients were lysed for analysis of RANKL concentration in synovial exosomes. The levels of RANKL were measured using sandwich enzyme-linked immunosorbent assay (ELISA). Data are presented as the mean ± SEM. * *p* < 0.05 vs. disease control, AS. (**B**) CD9 or anti-RANKL antibodies-coated magnetic beads and stained with Exo-FITC. The expression of CD9 and RANKL are presented by flow cytometric histograms.

We identified the molecules of synovial exosomes that induce osteoclastogenesis. We assessed RANKL levels in synovial exosomes, and it was expected to be one of the target exosomal molecules. RANKL levels were significantly higher in lysed exosomes from RA patients (84.29 ± 16.55 pg/mL, *p* = 0.035) than in those from AS patients (16.66 ± 16.66 pg/mL (Figure 3A). Furthermore, flow cytometric analysis of exosomal surface expression of RANKL (Figure 3B) revealed the presence of exosomal surface protein, CD9 [35], and their CD9 expression levels were similar in exosomes from RA and AS patients. RANKL expression was also detected on the surface of synovial exosomes and was much higher in exosomes of RA patients than in those of AS patients. These data suggest that RANKL is expressed on the surface of RA synovial exosomes and may contribute to osteoclast differentiation.

There was no direct correlation between RANKL levels in RA synovial exosomes and osteoclast differentiation (data not shown), probably owing to the limited number of samples in our experiment. Furthermore, other molecules, such as exosomal miRNAs, could contribute to various stages of osteoclastogenesis. 

## 4. Discussion

Synovial fluid in IA patients contains abundant inflammatory cytokines and immune cells. High levels of inflammatory cytokines, such as IL-1β, TNF-α, and proteolytic enzymes, enhance osteoclast differentiation and stimulate various cells in the synovium, such as synovial fibroblasts and leukocytes [2,3]. Furthermore, synovial fluid-derived exosomes play immuno-stimulatory roles in IA [17,36,37,38]; however, their role in osteoclast differentiation was unknown. Bone remodeling is regulated by osteoclasts and osteoblasts, and skeletal remodeling follows different patterns following the onset of various types of IA [4,5,6]. However, the molecular mechanisms, including factors originating from each type of IA, resulting in differential outcomes of skeletal remodeling, are unclear. In the present study, we verified the characteristics of exosomes isolated from synovial fluid from patients with various types of IA and evaluated their effects on human osteoclast differentiation.

In the present study, the number of exosomes in inflammatory arthritis, such as those in RA and AS patients, was significantly higher than those in non-inflammatory arthritis. Not only are there abundant activated immune cells in the synovium of inflammatory arthritis [2] but these immune cells can release numerous exosomes into the joint space and may communicate via their exosomal signals. Furthermore, our results suggest that increased exosomal levels in synovial fluid may reflect a high pro-inflammatory burden, and analysis of synovial exosomes may be used to evaluate the degree or quality of joint inflammation. 

Most of all, the present study focused on showing synovial exosomes in IA patients playing an important role in human osteoclastogenesis. Synovial exosomes from RA patients had a high osteoclastogenic potential even in the absence of essential growth factors, such as M-CSF and RANKL. Exosomes from non-erosive IA, including AS, and non-inflammatory arthritis, including OA, rarely induced osteoclast differentiation, and the number of osteoclasts was significantly lower than that in RA patients. The effect of synovial exosomes on osteoclast differentiation was not related to cell proliferation itself. Furthermore, the differences in the quality of synovial exosomes of RA and AS patients had different effects on osteoclastogenesis on adjusting the number of treated exosomes. The present results suggest that synovial exosomes of RA patients have high osteoclastogenic potential, which may partly explain the characteristic bony erosion observed in inflamed joints of RA patients, not frequently observed in other inflammatory arthritis. 

Recent data reported that exosomes containing miRNAs, mRNAs, proteins, and lipids are involved in intercellular communication via the transfer of their contents to recipient cells [14,15,16]. Therefore, we sought to identify synovial exosomal molecules that contribute to osteoclastogenesis. Exosomal RANKL levels are expected to be key targets because the addition of RA exosome on cultured monocyte induced osteoclast formation even without soluble RANKL itself. RANKL expression on the exosomal membrane and concentration of RANKL in lysed exosomes were higher in exosomes of RA patients than in those of AS patients. Although we did not observe a direct correlation between exosomal RANKL levels and bone erosion scores in RA patients, we showed that synovial exosomes of RA patients contain high levels of RANKL, which could effectively contribute to characteristic bony erosion in RA. Several studies reported that some miRNAs, such as miR-148a, mir-223 mir-21, and miR-31, regulate osteoclastogenesis [39,40,41,42]. We did not compare the quality and levels of miRNAs in the various types of IA in the present study; hence, further investigation is required to analyze miRNA profiles within exosomes of IA patients and assess their relevance with osteoclastogenesis.

In conclusion, the number of synovial exosomes may vary in accordance with various types of IA, each having a different osteoclastogenic potential. Synovial exosomes of RA patients may contain the disease-specific “synovial signature of osteoclastogenesis.”

## Data Availability

Data is contained within the article or Supplementary Materials.

## Supplementary Materials

The following are available online at https://www.mdpi.com/2073-4409/10/1/120/s1, Figure S1: Uptake of synovial exosomes by human macrophages. Figure S2: The effect of synovial exosomes on cell proliferation. Figure S3: Treatment of a fixed number of exosomes with human macrophages and the effect of synovial exosomes on human osteoclastogenesis.

## References

[1] Radner H., Ramiro S., Buchbinder R., Landewe R.B., van der Heijde D., Aletaha D. (2012). Pain management for inflammatory arthritis (rheumatoid arthritis, psoriatic arthritis, ankylosing spondylitis and other spondylarthritis) and gastrointestinal or liver comorbidity. Cochrane Database Syst. Rev..

[2] Feldmann M., Brennan F.M., Maini R.N. (1996). Role of cytokines in rheumatoid arthritis. Annu. Rev. Immunol..

[3] Miyasaka N., Sato K., Goto M., Sasano M., Natsuyama M., Inoue K., Nishioka K. (1988). Augmented interleukin-1 production and HLA-DR expression in the synovium of rheumatoid arthritis patients. Possible involvement in joint destruction. Arthritis Rheum..

[4] Goldring S.R., Purdue P.E., Crotti T.N., Shen Z., Flannery M.R., Binder N.B., Ross F.P., McHugh K.P. (2013). Bone remodelling in inflammatory arthritis. Ann. Rheum. Dis..

[5] Schett G. (2007). Joint remodelling in inflammatory disease. Ann. Rheum. Dis..

[6] Appel H., Loddenkemper C., Miossec P. (2009). Rheumatoid arthritis and ankylosing spondylitis—pathology of acute inflammation. Clin. Exp. Rheumatol..

[7] McQueen F.M., Doyle A., Reeves Q., Gao A., Tsai A., Gamble G.D., Curteis B., Williams M., Dalbeth N. (2014). Bone erosions in patients with chronic gouty arthropathy are associated with tophi but not bone oedema or synovitis: New insights from a 3 T MRI study. Rheumatology (Oxford).

[8] Nakashima T., Takayanagi H. (2009). Osteoimmunology: Crosstalk between the immune and bone systems. J. Clin. Immunol..

[9] Azuma Y., Kaji K., Katogi R., Takeshita S., Kudo A. (2000). Tumor necrosis factor-alpha induces differentiation of and bone resorption by osteoclasts. J. Biol. Chem..

[10] Pfeilschifter J., Chenu C., Bird A., Mundy G.R., Roodman G.D. (1989). Interleukin-1 and tumor necrosis factor stimulate the formation of human osteoclastlike cells in vitro. J. Bone Miner. Res..

[11] Mabilleau G., Sabokbar A. (2009). Interleukin-32 promotes osteoclast differentiation but not osteoclast activation. PLoS ONE.

[12] Mun S.H., Ko N.Y., Kim H.S., Kim J.W., Kim D.K., Kim A.R., Lee S.H., Kim Y.G., Lee C.K., Lee S.H. (2010). Interleukin-33 stimulates formation of functional osteoclasts from human CD14(+) monocytes. Cell. Mol. Life Sci..

[13] Boyle W.J., Simonet W.S., Lacey D.L. (2003). Osteoclast differentiation and activation. Nature.

[14] Saa P., Yakovleva O., de Castro J., Vasilyeva I., De Paoli S.H., Simak J., Cervenakova L. (2014). First demonstration of transmissible spongiform encephalopathy-associated prion protein (PrPTSE) in extracellular vesicles from plasma of mice infected with mouse-adapted variant Creutzfeldt-Jakob disease by in vitro amplification. J. Biol. Chem..

[15] Hannafon B.N., Ding W.Q. (2013). Intercellular communication by exosome-derived microRNAs in cancer. Int. J. Mol. Sci..

[16] Qin J., Xu Q. (2014). Functions and application of exosomes. Acta Pol. Pharm..

[17] Distler J.H., Pisetsky D.S., Huber L.C., Kalden J.R., Gay S., Distler O. (2005). Microparticles as regulators of inflammation: Novel players of cellular crosstalk in the rheumatic diseases. Arthritis Rheum..

[18] Song J., Kim D., Han J., Kim Y., Lee M., Jin E.J. (2015). PBMC and exosome-derived Hotair is a critical regulator and potent marker for rheumatoid arthritis. Clin. Exp. Med..

[19] György B., Szabo T.G., Turiak L., Wright M., Herczeg P., Ledeczi Z., Kittel A., Polgar A., Toth K., Derfalvi B. (2012). Im-proved flow cytometric assessment reveals distinct microvesicle (cell-derived microparticle) signatures in joint diseases. PLoS ONE.

[20] Raimondi L., De Luca A., Amodio N., Manno M., Raccosta S., Taverna S., Bellavia D., Naselli F., Fontana S., Schillaci O. (2015). Involvement of multiple myeloma cell-derived exosomes in osteoclast differentiation. Oncotarget.

[21] Taverna S., Pucci M., Giallombardo M., Di Bella M.A., Santarpia M., Reclusa P., Gil-Bazo I., Rolfo C., Alessandro R. (2017). Am-phiregulin contained in NSCLC-exosomes induces osteoclast differentiation through the activation of EGFR pathway. Sci. Rep..

[22] Adamopoulos I.E., Danks L., Itonaga I., Locklin R.M., Sabokbar A., Ferguson D.J., Athanasou N.A. (2006). Stimulation of osteoclast formation by inflammatory synovial fluid. Virchows Arch..

[23] Koch A.E., Kunkel S.L., Burrows J.C., Evanoff H.L., Haines G.K., Pope R.M., Strieter R.M. (1991). Synovial tissue macrophage as a source of the chemotactic cytokine IL-8. J. Immunol..

[24] Lettesjö H., Nordstrom E., Strom H., Nilsson B., Glinghammar B., Dahlstedt L., Moller E. (1998). Synovial fluid cytokines in patients with rheumatoid arthritis or other arthritic lesions. Scand. J. Immunol..

[25] Yarilina A., Xu K., Chen J., Ivashkiv L.B. (2011). TNF activates calcium-nuclear factor of activated T cells (NFAT)c1 signaling pathways in human macrophages. Proc. Natl. Acad. Sci. USA.

[26] Fuller K., Murphy C., Kirstein B., Fox S.W., Chambers T.J. (2002). TNFalpha potently activates osteoclasts, through a direct action independent of and strongly synergistic with RANKL. Endocrinology.

[27] Wei S., Kitaura H., Zhou P., Ross F.P., Teitelbaum S.L. (2005). IL-1 mediates TNF-induced osteoclastogenesis. J. Clin. Invest.

[28] Tanabe N., Maeno M., Suzuki N., Fujisaki K., Tanaka H., Ogiso B., Ito K. (2005). IL-1 alpha stimulates the formation of osteoclast-like cells by increasing M-CSF and PGE2 production and decreasing OPG production by osteoblasts. Life Sci..

[29] Liu X.H., Kirschenbaum A., Yao S., Levine A.C. (2006). The role of the interleukin-6/gp130 signaling pathway in bone metabolism. Vitam. Horm..

[30] Arnett F.C., Edworthy S.M., Bloch D.A., McShane D.J., Fries J.F., Cooper N.S., Healey L.A., Kaplan S.R., Liang M.H., Luthra H.S. (1988). The American Rheumatism Association 1987 revised criteria for the classification of rheumatoid arthritis. Arthritis Rheum..

[31] Savina A., Vidal M., Colombo M.I. (2002). The exosome pathway in K562 cells is regulated by Rab11. J. Cell Sci..

[32] Nguyen D.G., Booth A., Gould S.J., Hildreth J.E. (2003). Evidence that HIV budding in primary macrophages occurs through the exosome release pathway. J. Biol. Chem..

[33] Minkin C. (1982). Bone acid phosphatase: Tartrate-resistant acid phosphatase as a marker of osteoclast function. Calcif. Tissue Int..

[34] Reeve J.L., Zou W., Liu Y., Maltzman J.S., Ross F.P., Teitelbaum S.L. (2009). SLP-76 couples Syk to the osteoclast cytoskeleton. J. Immunol..

[35] Andreu Z., Yanez-Mo M. (2014). Tetraspanins in extracellular vesicle formation and function. Front Immunol..

[36] Berckmans R.J., Nieuwland R., Kraan M.C., Schaap M.C., Pots D., Smeets T.J., Sturk A., Tak P.P. (2005). Synovial microparticles from arthritic patients modulate chemokine and cytokine release by synoviocytes. Arthritis Res. Ther..

[37] Messer L., Alsaleh G., Freyssinet J.M., Zobairi F., Leray I., Gottenberg J.E., Sibilia J., Toti-Orfanoudakis F., Wachsmann D. (2009). Microparticle-induced release of B-lymphocyte regulators by rheumatoid synoviocytes. Arthritis Res. Ther..

[38] Knijff-Dutmer E.A., Koerts J., Nieuwland R., Kalsbeek-Batenburg E.M., van de Laar M.A. (2002). Elevated levels of platelet micro-particles are associated with disease activity in rheumatoid arthritis. Arthritis Rheum..

[39] Sugatani T., Hruska K.A. (2009). Impaired micro-RNA pathways diminish osteoclast differentiation and function. J. Biol. Chem..

[40] Sugatani T., Vacher J., Hruska K.A. (2011). A microRNA expression signature of osteoclastogenesis. Blood.

[41] Mizoguchi F., Murakami Y., Saito T., Miyasaka N., Kohsaka H. (2013). miR-31 controls osteoclast formation and bone resorption by targeting RhoA. Arthritis Res. Ther..

[42] Cheng P., Chen C., He H.B., Hu R., Zhou H.D., Xie H., Zhu W., Dai R.C., Wu X.P., Liao E.Y. (2013). miR-148a regulates osteoclastogenesis by targeting V-maf musculoaponeurotic fibrosarcoma oncogene homolog B. J. Bone Miner. Res..

