# Supplementary material for: Role of Synovial Exosomes in Osteoclast Differentiation in Inflammatory Arthritis
Source: Cells. 2021 Jan 10;10(1):120. doi: 10.3390/cells10010120 (PMC7827682; doi:10.3390/cells10010120)
Supplement: Supplementary file 1 [file cells-10-00120-s001.pdf]

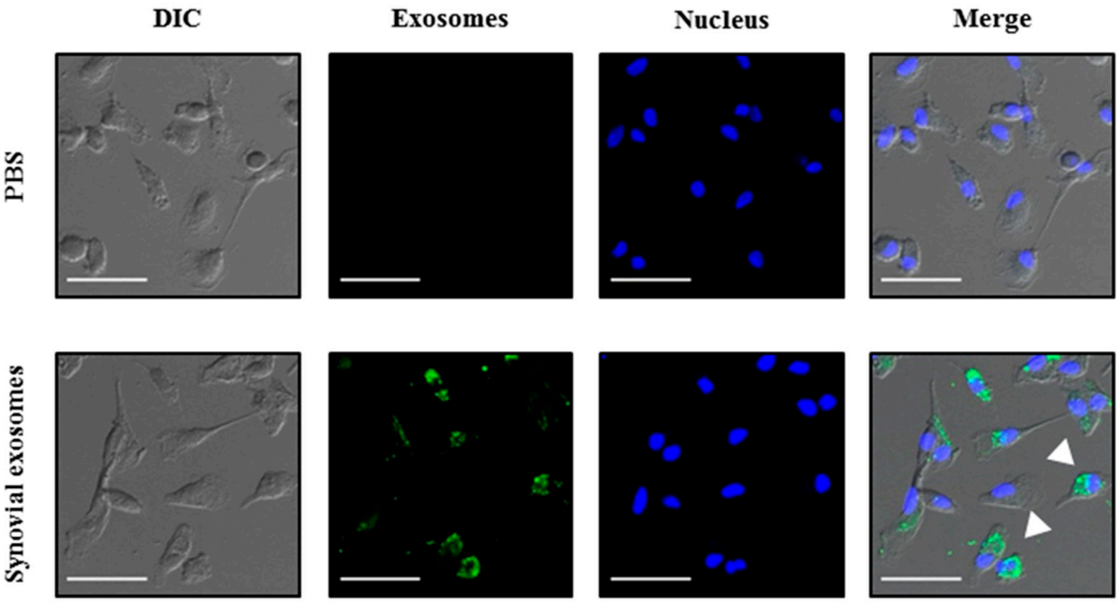

Figure S1. Uptake of synovial exosomes by human macrophages. Human macrophages were incubated with CFSE-labeled exosomes or PBS (negative control) at 37 °C. After 3 h of incubation, cells were fixed and nuclei were stained using Hoechst 33258. Images were analyzed using confocal microscope. The white arrow indicates cellular uptake of synovial exosomes. Scale bar = 40 μm. DIC = differential interference contrast.

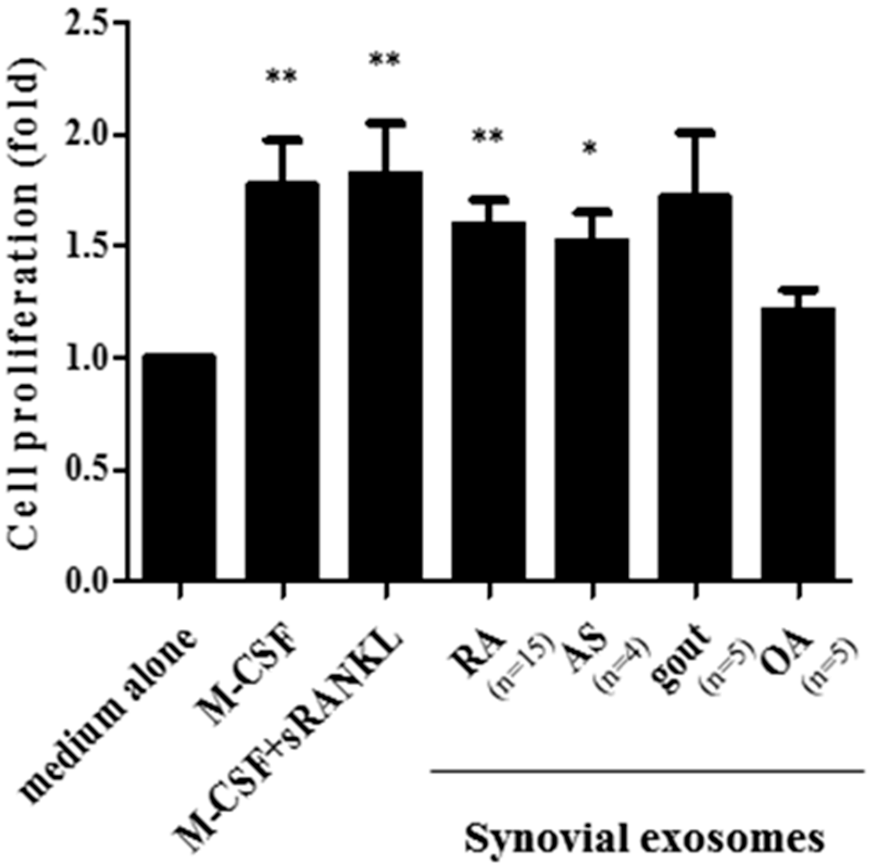

Figure S2. The effect of synovial exosomes on cell proliferation. Human macrophages differentiated from $CD14^{+}$ monocyte were treated with 20 ng/mL of M-CSF +40 ng/ml of sRANKL (positive control) or with 10% synovial exosomes, respectively. Exosomes were isolated from same volume of synovial fluid with RA, AS, gout, and OA patients. After 48 h of incubation, cell proliferation was measured using CCK-8. Data are presented as a fold change of cell proliferation compared to medium alone (negative control) and presented as the mean ± SEM. * $p < 0.05$ vs. medium alone, ** $p < 0.01$ vs. medium alone.

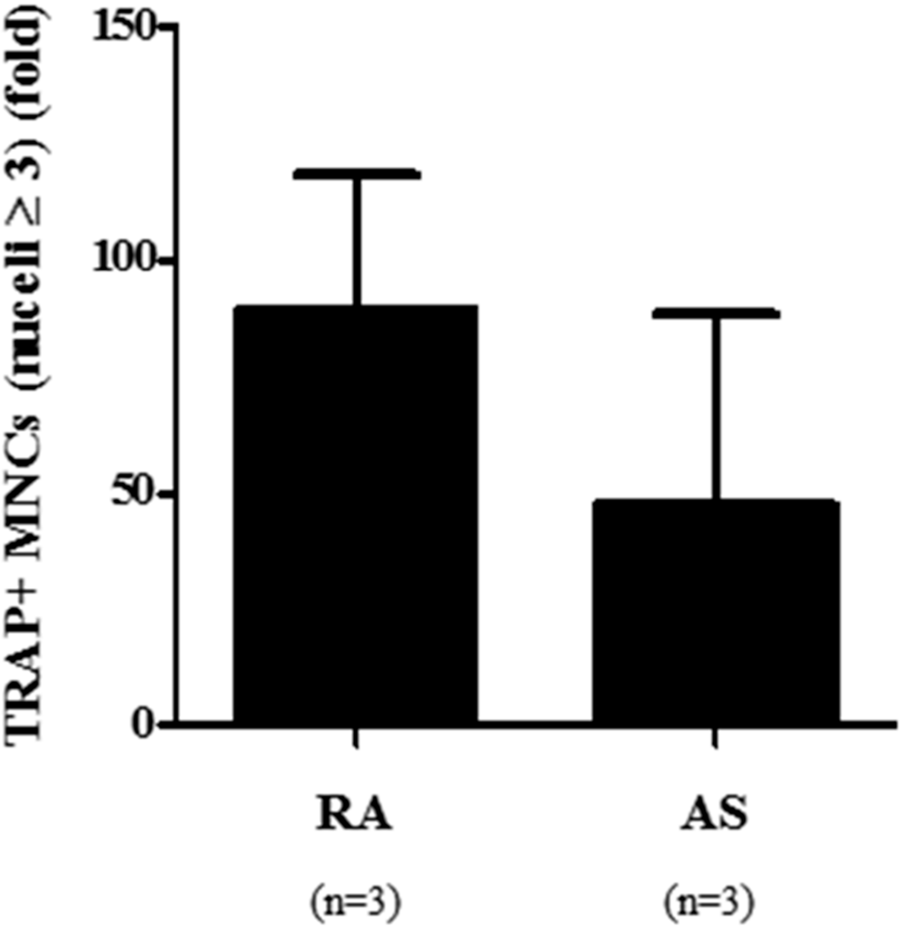

Figure S3. Treatment of a fixed number of exosomes with human macrophages and the effect of synovial exosomes on human osteoclastogenesis. Human macrophages were treated with a fixed number of exosomes isolated from RA or AS patients. After 9–10 d of incubation, cells were stained for TRAP expression and TRAP-positive multinucleated cells (MNCs) were counted. Data are presented as a fold change of osteoclast number compared to medium alone (negative control). Data are presented as the mean ± SEM.
